# Type D personality is associated with increased metabolic syndrome prevalence and an unhealthy lifestyle in a cross-sectional Dutch community sample

**DOI:** 10.1186/1471-2458-10-714

**Published:** 2010-11-19

**Authors:** Paula MC Mommersteeg, Nina Kupper, Johan Denollet

**Affiliations:** 1 CoRPS - Center of Research on Psychology in Somatic diseases, Dept. of Medical Psychology, Tilburg University, The Netherlands

## Abstract

**Background:**

People with Type D-Distressed-personality have a general tendency towards increased negative affectivity (NA), while at the same time inhibiting these emotions in social situations (SI). Type D personality is associated with an increased risk of adverse outcomes in patients with cardiovascular disease. Whether Type D personality is a cardiovascular risk factor in healthy populations remains to be investigated. In the present study, the relations between Type D personality and classical cardiovascular risk factors, i.e. metabolic syndrome and lifestyle were investigated in a Dutch community sample.

**Methods:**

In a cross-sectional study 1592 participants were included, aged 20-80 years. Metabolic syndrome was defined by self-report, following the International Diabetes Federation-IDF-guidelines including an increased waist circumference, dyslipidemia, hypertension, and diabetes. In addition lifestyle factors smoking, alcohol use, exercise and dietary habits were examined. Metabolic syndrome prevalence was stratified by Type D personality (a high score on both NA and SI), lifestyle and confounders age, gender, having a partner, higher education level, cardiac history, family history of cardiovascular disease.

**Results:**

Metabolic syndrome was more prevalent in persons with a Type D personality (13% vs. 6%). Persons with Type D personality made poorer lifestyle choices, adhered less to the physical activity norm (OR = 1.5, 95%CI = 1.1-2.0, *p *= .02), had a less varied diet (OR = 0.50, 95%CI = 0.40-0.70, *p *< .0005), and were less likely to restrict their fat intake (OR = 0.70, 95%CI = 0.50-0.90, *p *= .01). Type D personality was related to a twofold increased risk of metabolic syndrome (OR = 2.2, 95%CI = 1.2-4.0, *p *= .011), independent of lifestyle factors and confounders.

**Conclusions:**

Type D personality is related to an increased prevalence of metabolic syndrome and unhealthy lifestyle, which suggests both behavioral and biological vulnerability for development of cardiovascular disorders and diabetes.

## Background

Type D (*Distressed*) personality has been associated with an increased risk of adverse cardiac events in patients with a cardiovascular condition [1-4]. Both behavioral (e.g. poor consultation behavior) and biological (e.g. cortisol hyperactivity, cardiovascular hyper-reactivity, immune factors) mechanisms have been suggested [5,6]. Individuals with a Type D personality have the tendency to experience increased negative emotions and inhibit these emotions in social situations, because of fear of rejection or disapproval. Type D personality is a stable and heritable character trait rather than a consequence of cardiac disease [7-9], thus a pre-existing vulnerability profile may be present in persons with Type D personality.

The metabolic syndrome and an unhealthy lifestyle represent standard risk factors for cardiovascular disease and diabetes [10]. Metabolic syndrome refers to a cluster of risk factors, including increased central fat deposition, glucose intolerance or insulin resistance, dyslipidemia and hypertension, which progressively contribute to the atherosclerotic process, consequent cardiovascular disease and diabetes development [11]. Adverse lifestyle factors such as smoking, excessive alcohol consumption, an unhealthy diet and insufficient physical exercise are also related to an increased risk of cardiovascular conditions [12-14].

Previous studies in patients with cardiovascular disease have investigated the relation between Type D personality and components of the metabolic syndrome. Studies in CAD patients observed no differences in hypertension, hypercholesterolemia or diabetes mellitus as a function of Type D personality [15,16]. However, Type D personality was more prevalent in patients with hypertension (53%) as compared to healthy individuals (19%) [17]. Although these studies do not directly point to an increased risk for metabolic syndrome components in persons with Type D personality, these studies were all done in patients already diagnosed with cardiovascular disease.

There are several studies that link Type D personality to unhealthy lifestyle factors. A recent study pointed out that Type D personality was much more prevalent in (otherwise healthy) men with a sedentary lifestyle (45%) as opposed to men that exercised regularly (14%) [18], while another study revealed that healthy students with a Type D personality demonstrated poorer health behaviors such as eating sensibly, spending time outdoors, and getting regular medical check-ups, as compared to their non-Type D counterparts [19]. Smoking has been evaluated in cardiovascular patients, showing that Type D patients smoke as much as non-Type D patients [20,21].

Type D personality may be viewed as a relatively novel risk factor, although it has been around since the early nineties of the previous century. One important characteristic of a novel risk marker to become a well-accepted risk factor is that it should add independent information of risk or prognosis [22]. While Type D personality has been repeatedly associated with poor prognosis and increased risk of morbidity and mortality in cardiac patients (for a review see [23]), no previous study has shown Type D personality to be associated with increased cardiovascular risk in healthy populations.

The current study therefore examined whether Type D personality is related to an increased prevalence of metabolic syndrome and an unhealthy life style in a large community sample.

## Methods

### Participants

The sample comprised a convenient selection of 1592 participants from the Dutch general population residing in the Southern provinces of the Netherlands (population of approx. 4 million). Data were collected between October, 1, 2008 and December, 15, 2008. Quota sampling was applied to ensure equal representation of each sex and age group between 20 and 80 years; e.g., the number of men between 20 and 29 years was equal to the number of women between 60 and 69 years. Research assistants were responsible for distributing the questionnaires and were instructed to collect an equal amount of questionnaires from each age and sex sub cohort, without further specification of educational or income level. Participants were approached personally or by phone. After explaining the purpose of the study, participants received an informed consent form and a questionnaire, which were returned to the research assistants in closed envelopes. The questionnaires were entered into the database by others, guaranteeing anonymity. Returned questionnaires did not contain any explicit identifiers (i.e., names) but rather, were coded by number for purposes of data collection tracking. Approval for this study was obtained from a local ethics committee at Tilburg University (protocol number: 2006/1101).

### Type D personality

Type D, or Distressed personality is a combination of Negative Affectivity (NA) and Social Inhibition (SI). NA is defined as the tendency to experience negative emotional states across time and situations, comprising dysphoria, feelings of tension, and worry. SI involves the tendency to inhibit the expression of emotions, thoughts and behaviors in social interaction, due to anticipation of negative reactions from others [5]. The presence of both traits is an essential characteristic of Type D personality. The DS14 was used to assess Type D personality and consists of 14 items, 7 that assess the subcomponent NA and 7 assessing SI with a 0 to 4 Likert response scale. Persons are characterized with a Type D personality when scoring at least 10 on both subcomponents. The DS14 is a valid instrument, with high internal consistency (Cronbach's alphas NA: 0.87; SI: 0.87) and good test-retest reliability [7,17]. Cross-cultural validity has been established, as Type D personality has been validly and reliably assessed in multiple countries (e.g.,[24-27].

### Metabolic syndrome

In the present study, a self-report proxy based on the IDF-criteria has been used for defining metabolic syndrome presence [10]. These IDF-criteria include three out of five of the following criteria: an increased waist circumference (WC; European men >94 cm, European women >80 cm), raised triglycerides levels ≥ 150 mg/dL (1.7 mmol/L); reduced HDL cholesterol< 40 mg/dL in males and < 50 mg/dL in females, or treatment for this lipid abnormality; blood pressure > 130/85 mmHg or treatment of hypertension; glucose ≥ 100 mg/dL (5.6 mmol/l), or diagnosed with type 2 diabetes [10].

Waist circumference was measured by tape measure by participants themselves following instructions (in words as well as with a picture showing where exactly to measure waist and hip circumference), and reported in the questionnaire. Previous studies have shown good reliability of self-reported waist-circumference in comparison to examination by a trained professional [28-30]. There was a significant correlation between self-reported BMI and waist-circumference (r = 0.742, p < .001, N = 1229), and all participants with a BMI > 30 also met the criteria for increased waist circumference. Persons who did not fill out their waist-circumference, but who had a BMI >30 were also appointed to the increased risk group [10]. Further, several purpose-designed self-report questions asked for information on dyslipidemia (*"Has your cholesterol level been measured in the past three months?" With answer categories 'No', 'Yes, it was increased', 'Yes, it was normal'*), hypertension (1. *"Have you been diagnosed with hypertension? Answer categories: yes/no", 2. "Has your blood-pressure been measured in the past three months?" with answer categories 'No', 'Yes, my blood pressure was too high', 'Yes, my blood pressure was normal'*), and diabetes (*"Have you been diagnosed with diabetes? Answer categories: yes/no"*). When data was missing on either of above questions we classified participants as having dyslipidemia, hypertension and/or diabetes when they used statins to treat lipid abnormality, reported to use beta-blockers or ACE-inhibitors for hypertension or insulin for diabetes. As self-report measures were used in this study, a proxy measure of metabolic syndrome was calculated based on the above IDF criteria. We could not distinguish between HDL-cholesterol and triglyceride levels as total cholesterol levels were asked. Therefore metabolic syndrome was calculated based three-out-of-four, rather than three-out-of-five criteria of the IDF.

### Lifestyle factors

#### Exercise

The amount of physical activity was assessed in several ways. First, a question in the survey asked whether participants adhered to the Dutch guidelines for healthy physical activity [NNGB guideline; [31]]. This norm variable consisted of three categories, i.e., 100% adherence, 50% adherence and no adherence to the norm. Second, we specifically asked people whether they exercised regularly, and if so, to report frequency and duration per week per exercise. From this information we calculated the Dutch fit norm (exercising at least 20 minutes at least three occasions during the week), as well as the energy expenditure from sport activities by calculating the average MET (metabolic equivalent intensity) score [32] and the total amount of energy spent in sport activities in kilojoules.

#### Diet

Dietary habits were assessed by several purpose-designed questions asking whether participants ate a varied diet, and limited the amount of salt and fat in their food. For the logistic regression of metabolic syndrome, these three questions were aggregated into one variable in which 0 represents participants not adhering to all these healthier eating advices, 1 represents participants adhering to some of these advices and 2 represents participants adhering to all three advices. In addition, a food frequency questionnaire was presented that was adapted from the EPIC FFQ [33], by taking 27 food categories that were also present in the 2006 AHA diet and lifestyle recommendations [34]. Participants were asked to report their frequency of use of these 27 food categories (e.g. rice, bread, vegetables, fruits, white meat, red meat, fish, olive oil, butter, processed food, etc.) in times per day, per week, per month or per year, or never. We aggregated the food consumption information into three dietary patterns (i.e. cosmopolitan dietary pattern, a traditional pattern and a refined foods pattern), previously identified in the MORGEN study [35]. The cosmopolitan dietary pattern is characterized by a greater consumption of e.g. vegetables, white meats and less consumption of potatoes, while the traditional pattern is characterized by greater consumption of e.g. red meats, potatoes, and less consumption of e.g. soy products and fruits. The refined pattern is characterized by greater consumption of e.g. fried products, high-sugar beverages, snacks and less consumption of e.g., vegetables and whole-grain bread. Participants were allocated to one of these patterns when the majority of consumed foods belonged to one of the dietary patterns.

### Confounding variables

#### Cardiovascular disease

Genetic predisposition to cardiovascular disease is a potential confounder of metabolic syndrome [11]. We therefore asked participants whether they had family diagnosed/deceased with/from cardiovascular disease *(answer categories 'no', 'yes, first degree relatives', 'yes, second degree relatives, and 'yes, third degree relatives)' *The question on the family history of CAD was recoded into 1 = presence and/or mortality of a first degree relative, and 0 = other. We also asked whether participants themselves were diagnosed with cardiovascular disease (Q: "D*o you have established cardiovascular disease*" yes/no).

#### Educational level and social status score

Socio-demographic factors such as educational level, presence of a partner and social status have been associated with an increased risk of metabolic syndrome [11] and unhealthy lifestyles [11]. In addition to educational level, which was coded into 'higher educational level' (reporting at least college education) and 'lower educational level', social status scores were compared. Dutch national social status scores are available per postal code area and are based on national data on income, educational level and employment (http://www.scp.nl 'Statusscores' in SCP-Cahier 152 "Van hoog naar laag, van laag naar hoog" ISBN 90-5749-117-6). Social status was recoded into three groups; low, middle and high social status score.

### Statistical analysis

Chi-square tests were used to examine differences between Type D and non-Type D participants in categorical variables. In total 6 cases had missing info on Type D personality, thus the maximum sample size was 1592. In case of continuous variables, univariate general linear models (ANOVA) were used. To examine differences in exercise and dietary habits, we used chi-square tests in univariate analyses and multinomial regressions in multivariate analyses, while adjusting for the effects of age, gender, partner status and educational level.

To establish the association between Type D personality and metabolic syndrome prevalence, stepwise logistic regression was performed, in which Type D was entered first. In a second and third step, classic risk factors and lifestyle factors exercise and diet were entered. In post-hoc analyses, we reversed this logistic regression, entering Type D personality last, to examine changes in betas on the other variables, enabling the formulation of hypotheses regarding potential mediating mechanisms.

## Results

### Population characteristics

The prevalence of Type D personality was 13.3%. Type D personality was associated with female sex, being single, and lower education level (Table 1). There was a larger beer consumption in male non-Type D participants (MWU = 24536, *p *= .06) and a larger wine consumption in female non-Type D participants (MWU = 34289, *p *= .02) as compared to male and female Type Ds respectively. There were no differences in age, smoking, social status, presence of cardiovascular disease, or family history of CAD.

**Table 1:** Group characteristics stratified by Type D personality

		Total		Type D		non Type D		Test	
	N	%	*n*	%	*n*	%	*n*	X^2^/*F*	*p*
**Socio-demographic**									
Male sex	1591	*49.6*	*(789)*	**41.2**	(87)	**50.9**	(702)	**6.7**	**.009**
Age mean (SD)	1592	46.9	(16.1)	47.3	(16.6)	46.8	(16.0)	0.129	.720
With partner	1590	81	(1280)	**73.5**	(155)	**81.6**	(1125)	**7.69**	**.006**
Higher education	1573	31.9	(502)	**20**	(40)	**34**	(462)	**16.7**	**< .001**
Low social Status	1522	32.2	(490)	36.1	(73)	31.6	(417)	5.28	.072
**Cardiovascular risk factors**									
Smoking (yes)	1582	24.7	(390)	25.7	(54)	24.5	(336)	0.147	.701
Alcohol use (yes)*	1586	83.8	(1290)	**76.1**	**(153)**	**85.0**	**(1137)**	**10.1**	**.001**
Cardiovascular disease	1549	9	(140)	10.3	(21)	8.8	(119)	0.40	.486
First degree family history of CAD (presence and/or mortality)	1522	30.9	(470)	35.5	(71)	30.2	(399)	2.30	.129

* at least 1 alcoholic consumption per week

### Metabolic syndrome

In total 114 persons (7.3%) met the criteria for metabolic syndrome (Table 2). There was an increased number of participants with Type D personality who were characterized by the metabolic syndrome criteria; 13% in the Type D versus 6.4% in the non-Type D group. When observing the individual components of metabolic syndrome, an increased number of Type D participants showed lipid abnormality or hypertension. There was no difference in waist-circumference or obesity between the Type D and the non-Type D group, or in the number of participants reporting diabetes.

**Table 2:** Metabolic syndrome stratified by Type D personality

		Total		Type D		non Type D		Test	
	*N*	%	*n*	%	*n*	%	*n*	X^2^	*p*
Metabolic syndrome	1572	7.3	(114)	**13**	(27)	**6.4**	(87)	11.7	**.001**
**Components**									
Waist-circumference or BMI >30*	1557	55.6	(865)	51.9	(107)	56.1	(758)	1.26	.262
Lipid abnormality^†^	1579	7.7	(122)	**12.4**	(26)	**7**	(96)	7.36	**.007**
Hypertensive^‡^	1584	13.6	(215)	**18.1**	(38)	**12.9**	(177)	4.22	**.040**
Diabetic^§^	1542	4.7	(73)	5.9	(12)	4.6	(61)	0.69	.407

*Waist circumference = > 94 for men, and = > 80 for women, if missing: BMI > 30

^†^Lipid abnormality = high cholesterol in the past three months or statin use

^‡^Hypertensive = high blood pressure in the past three months, diagnosed with hypertension, or use of beta-blockers or ACE-inhibitors.

^§^Diabetic = diabetes type 2

**Figure 1:**
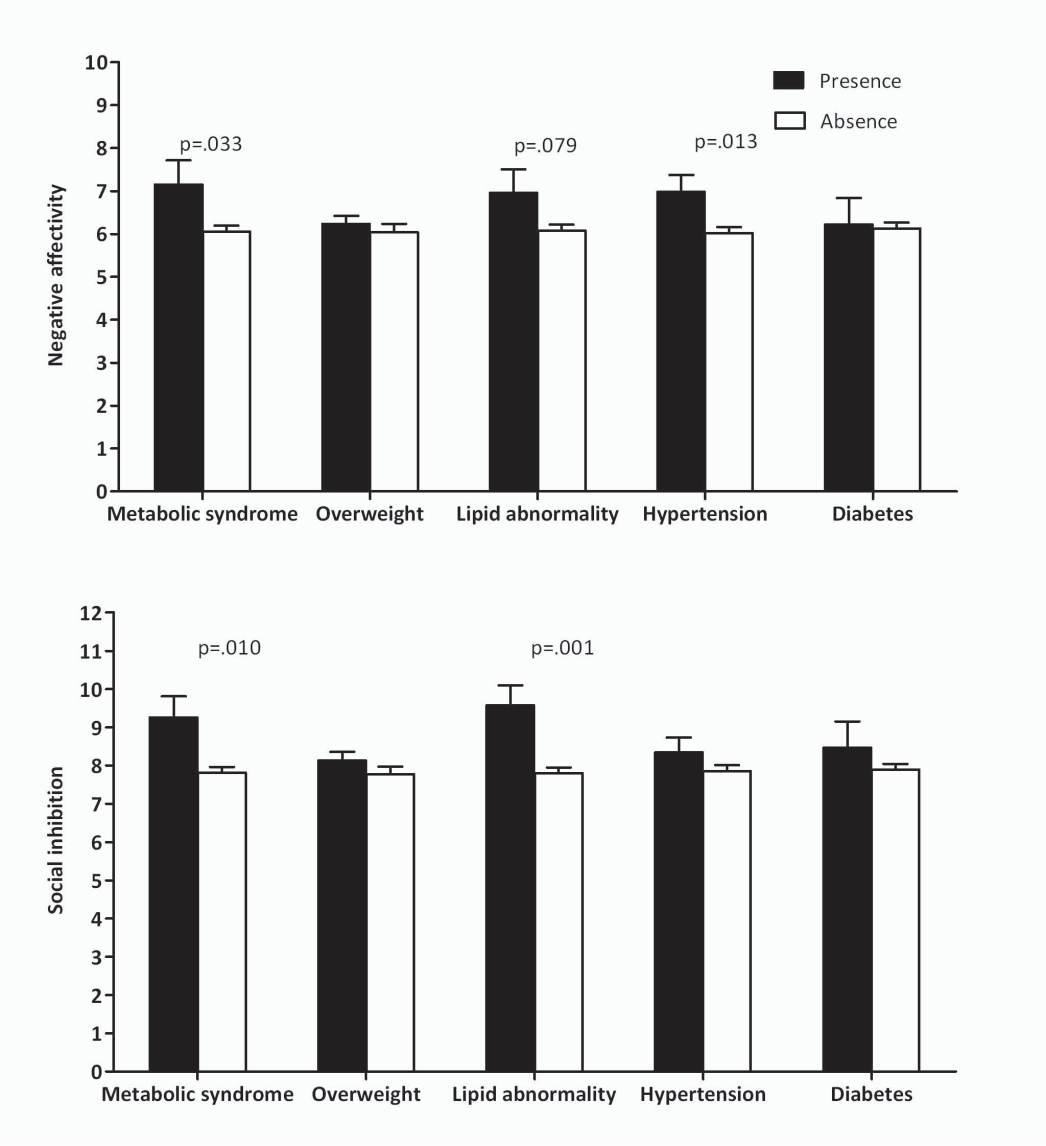
**Type D subscales negative affectivity and social inhibition stratified by metabolic syndrome and its components**. NA (above) and SI (below) scales stratified by presence and absence of metabolic syndrome and its components overweight, lipid abnormality, hypertension and diabetes. People who report metabolic syndrome have significantly higher negative affectivity and social inhibition scores. METS components show a significant higher level of NA for hypertension (*F*(1,1583) = 6.2), and a trend for lipid abnormality (*F*(1,1578) = 3.1), and a higher score for SI on lipid abnormality (*F*(1,1578) = 10.8). Mean and SEM are shown.

Post-hoc analyses compared average NA and SI scores as a function of the metabolic syndrome and its components (Figure 1). Significantly higher scores of NA and SI were reported in the presence of metabolic syndrome. NA was significantly associated with hypertension and a tendency toward lipid abnormality (Figure 1, top), and SI with lipid abnormality, but not hypertension, overweight or diabetes presence (Figure 1, below).

### Lifestyle factors

#### Exercise

Participants with a Type D personality less often adhered to the Dutch norm for healthy physical activity, as well as to the Dutch fit norm (Table 3). However, Type D participants that exercised regularly did this as intensive as non-Type D participants, as becomes clear from the average MET score and the total energy expenditure (Table 3).

**Table 3:** Physical activity stratified by Type D personality

		Total (*n *= 1577)	Type D (*n *= 209)	Non-Type D (*n *= 1369)	Univariate		**Multivariate**^†^	
					**χ^2^**	***p*-value**	**OR (95% CI)**	***p*-value**

Dutch healthy physical activity norm	No	10%	12%	10%	6.85	.03	1.6 (1.0-2.5)	.06
	Sometimes	29%	35%	28%			1.5 (1.1-2.0)	.02
	Yes	61%	52%	62%			Reference category	
Dutch fit norm	No	43%	51%	42%	8.95	.01	1.6 (1.1-2.2)	.02
	Partly	24%	24%	24%			1.3 (0.9-2.0)	.20
	Yes	33%	25%	35%			Reference category	
		
		***n *= 656**	***n *= 79**	***n *= 627**	***T***	***p*-value**	***F***	***p*-value**
		
Average MET score*		6.2 (1.9)	6.0 (1.9)	6.3 (1.9)	1.14	.26	0.103	.75
Average energy expenditure* (kJ)		1702 (1134)	1565 (1239)	1719 (1124)	1.07	.29	0.096	.76

* Only for those people who exercise (n = 656)

^†^Adjusted for age, gender, partner status, higher education

MET = average metabolic energy rate for sport activities

In multivariate analyses, we examined whether Type D personality was still related to less frequent physical activity, when controlling for the covariates gender, categorized age, partner status, and education. Results showed that Type D participants had an increased chance of not exercising regularly, and not being physically active often enough (Table 3). Age was a significant covariate of adhering to the Dutch fit norm, with higher age having an increased chance of not exercising enough (OR = 1.3 (95%CI = 1.1-1.6), *p *= .007), or not exercising at all (OR = 1.6 (95%CI = 1.3-1.8), *p *< .001). Sex differences were found for the average MET score and total energy expenditure for exercise activities in participants that exercise, with men performing more demanding sports activities (*F*(1,711) = 15.78, *p *= < .001) and exercising more intensively (*F*(1,653) = 102.76, *p *< .001).

#### Dietary habits

Univariate analyses showed that Type D individual had a less varied diet (χ^2 ^= 19.944, *p *< .001), and were less likely to restrict the amount of fat (χ^2 ^= 4.900, *p *= .03) as compared to non-Type D individuals (Table 4). There was no significant difference in salt restriction. After adjustment for the covariates age, gender, partner status and education, Type D personality remained significantly associated with a less varied diet and increased intake of fats.

**Table 4:** Dietary habits stratified by Type D personality

	Total	Type D	Non-Type D	Univariate		Multivariate*	
	**N = 1582**	**n = 209**	**n = 1373**	**χ^2^**	***p*-value**	**OR**	***p*-value**

Varied diet (yes)	75.4%	63.0%	77.3%	19.94	< .001	0.5 (0.4-0.7)	< .001
Restrict fats (yes)	60.6%	53.6%	61.6%	4.89	.03	0.7(0.5-0.9)	.01
Restrict salt (yes)	56.4%	53.1%	56.9%	1.05	.31	0.8 (0.6-1.1)	.15
Cosmopolitan	29.5%	25.8%	30.1%	3.41	.18	Reference category	
Traditional	54.4%	54.0%	54.4%			1.3 (0.9-1.8)	.23
Refined	16.1%	20.2%	15.5%			1.5 (1.0-2.5)	.08

Note: Information on the different dietary patterns was present for 1505 subjects

* Adjusted for age, gender, partner status, higher education

Individuals were more likely to eat a varied diet and to restrict the amount of salt and fats in their diet when they had a partner (OR = 1.3-1.8; *p *< .03), and belonged to older aged subgroups: 45-65 years of age: OR = 1.4-1.6 p < .02; aged over 65: OR = 1.5-2.7; *p *< .02). Men were less likely to eat a varied diet and to restrict the amount of salt and fats in their diet (OR = 0.6-0.8; *p *< .02). Participants with higher educational levels (at least college education) were more likely to eat a varied diet (OR = 1.4, *p *= .009) but did not differ from less educated participants in the restriction of salt and fat.

The three food consumption patterns identified in the MORGEN study [35] were present in our sample, and comprised respectively 29% (cosmopolitan), 55% (traditional) and 16% (refined) of our dataset. These dietary patterns did not associate with Type D personality in uni- and multivariate analyses (Table 4). Women and higher educated participants were less likely to adhere to a traditional (OR = 0.6-0.7; *p *< .01) or refined dietary pattern (OR = 0.4-0.5, *p *< .001). Older aged participants were more likely to adhere to the traditional dietary pattern (OR = 1.2, *p *= .03), and less likely to have a refined food consumption pattern (OR = 0.5, *p *< .001).

### Multivariate logistic regression of metabolic syndrome

In a stepwise manner the presence of metabolic syndrome was related to Type D personality, psychosocial and cardiovascular risk factors, and lifestyle factors (Table 5). In the first step, Type D personality was entered, controlling for gender, age (3 categories), living together with a partner, and higher education level. People with Type D personality had an increased risk for metabolic syndrome (Table 5). Type D remained significantly related to an increased prevalence for metabolic syndrome after controlling for presence of cardiovascular disease, family history of CAD, smoking, and alcohol use. In the final step, lifestyle factors representing adherence to the Dutch eating and exercise guidelines were introduced. Lifestyle factors were not significantly related to metabolic syndrome, and hence Type D personality remained significantly related to metabolic syndrome presence (OR = 2.2, 95% CI = 1.2-4.0, *p *= .011). Other significant contributors to metabolic syndrome prevalence in the final model were (older) age, with older people having an increased odds, living together with a partner (OR = 4.2, 95% CI = 1.7-10.6, *p *= .002), and finally presence of cardiovascular disease (OR = 6.2, 95% CI = 3.6-10.8, *p *< .001).

**Table 5:** Logistic regression of metabolic syndrome

	Step 1				Step 2				Step 3			
		**95% CI for OR**				**95% CI for OR**				**95% CI for OR**		
	**OR**	**Lower**	**Upper**	**Sig**.	**OR**	**Lower**	**Upper**	**Sig**.	**OR**	**Lower**	**Upper**	**Sig**.

Type D (yes)	**2.31**	**1.32**	**4.05**	**.003**	**2.17**	**1.19**	**3.97**	**.011**	**2.19**	**1.20**	**4.02**	**.011**
Gender (male)	1.16	0.74	1.84	.514	1.02	0.63	1.67	.933	1.03	0.63	1.68	.920
Age < 45 years				.000				.000				.000
45-65 years	**12.5**	**4.94**	**31.6**	**.000**	**9.86**	**3.85**	**25.2**	**.000**	**9.72**	**3.79**	**24.9**	**.000**
>65 years	**27.8**	**10.6**	**73.1**	**.000**	**13.3**	**4.84**	**36.5**	**.000**	**13.2**	**4.78**	**36.3**	**.000**
With partner (yes)	**3.58**	**1.50**	**8.56**	**.004**	**4.36**	**1.75**	**10.9**	**.002**	**4.24**	**1.70**	**10.6**	**.002**
College Education (yes)	0.91	0.54	1.54	.737	0.90	0.52	1.56	.713	0.90	0.52	1.57	.714
CVD (yes)					**6.32**	**3.69**	**10.8**	**.000**	**6.25**	**3.60**	**10.8**	**.000**
CVD family history (yes)					1.12	0.69	1.81	.657	1.13	0.69	1.83	.635
Smoking (yes)					0.81	0.44	1.48	.495	0.82	0.45	1.49	.508
Alcohol use > 1 (yes)					0.72	0.41	1.27	.250	0.71	0.40	1.25	.237
Healthy exercise norm (not adhering)									reference			.921
									1.17	0.52	2.65	.705
									0.99	0.57	1.74	.982
Diet guidelines (not adhering)									reference			.729
									0.74	0.34	1.62	.452
									1.00	0.59	1.69	.999
-2LL of the model started at 605.65	570.35				522.53				521.80			

CVD = Cardiovascular Disease

N = 1400 cases in the analysis

In post-hoc analyses, we reversed the stepwise inclusion of variables into the logistic regression so that first, risk factors and lifestyle factors were entered, and then Type D personality so that from the changes in betas from the other variables hypotheses may be derived on potential mediating mechanisms. This resulted in the observation that none of the betas changed significantly when adding Type D personality, suggesting that additional variance in metabolic syndrome is explained by Type D personality classification.

## Discussion

The findings of the present study show that people with Type D personality had a twofold increased risk of having metabolic syndrome, independent of established metabolic syndrome risk factors such as age, gender, having a partner, education level, presence of CVD, family history of CVD, smoking, alcohol use, diet and exercise. The twice as large prevalence of metabolic syndrome in people with Type D may be attributed to the metabolic syndrome components dyslipidemia and hypertension, but not to waist-circumference or diabetes. Post-hoc analyses showed that higher levels of both NA and SI were associated with increased prevalence of metabolic syndrome. With respect to the individual metabolic risk markers, lipid abnormalities were associated with both NA and SI subcomponents, hypertension was associated with increased NA levels. This finding stresses the significance of high scores on both Type D subcomponents for the Type D personality construct.

There are no studies to date exploring the relation between Type D personality and metabolic syndrome. Recent studies on cardiac risk factors show a decreased prevalence of dyslipidemia in men with Type D personality, increased hypertension prevalence in women with Type personality, and no differences in cholesterol/HDL quotient, obesity, or diabetes prevalence [36]. Einvik and colleagues observed an increased BMI in persons with Type D personality, and higher triglyceride levels, but not other differences in cholesterol, blood pressure, waist/hip ratio, or fasting serum glucose [37]. Several psychological constructs related to negative affect, such as depression, hostility, and anger, have been found to be associated with metabolic syndrome [38-42]. These studies present cross-sectional evidence for a relationship in cardiac patients, e.g., a study by Vaccarino and colleagues showed an independent association of metabolic syndrome with depression in women with suspected coronary artery disease [39], while the Heart & Soul study showed significant univariate associations between depression, hostility and optimism-pessimism and metabolic syndrome prevalence in stable coronary artery disease patients [41]. An Australian study examined the association between depression and metabolic syndrome in the general population and found that metabolic syndrome was related to higher depression scores, but not anxiety or psychological distress [40]. In addition to the cross-sectional evidence, there is also some prospective evidence that depression, hostility, and anger predict increased risk for developing the metabolic syndrome [38]. Negative findings have been published as well, as Herva and colleagues, in a 31 year old cohort in Northern Finland, reported that no relation was observed between depression and the metabolic syndrome after controlling for covariates [43], and recently, a study in 1,212 elderly participants from the Longitudinal Aging Study Amsterdam reported the absence of a relation between major depression and the metabolic syndrome [42]. The significant relation between depression, hostility, and pessimism-optimism with metabolic syndrome in cardiac patients from the Heart and Soul study was no longer significant after controlling for socioeconomic status and health behaviors like physical activity, smoking, regular alcohol use, and BMI [41].

The current study observed lifestyle differences for Type D personality as well, i.e. persons with a Type D personality adhered less to the Dutch norm for healthy physical activity and the Dutch fit norm, but for the subgroup that did exercise, Type D personality was not related to the average metabolic energy rates or energy expenditure. Further, Type D persons less often ate a varied diet, and restricted the intake of fat to a lesser extent. There were no differences in salt restriction or diet category. However, exercise and dietary lifestyle factors did not affect the increased risk of metabolic syndrome associated with Type D personality. Similar findings have been observed in other studies: people with Type D personality had a somewhat less healthy diet, and were less physically active, or spend less time outdoors [19,36,37].

Primary intervention for metabolic syndrome involves lifestyle modification, weight management, diet and exercise changes. In healthy individuals previous studies have shown that poor dietary habits (less prudent food choices) [44] and low exercise are associated with some individual metabolic risk markers [45,46], as well as an increased risk of developing the metabolic syndrome [47]. Our results are not concurrent with these previous findings, as dietary habits and exercise did not explain additional variance in the logistic regression model. One explanation for this might be that many of the other variables in the model, i.e. Type D personality, age, BMI, educational level, smoking, presence of cardiovascular disease and eating a varied diet were all significantly associated with adherence to the Dutch norm for healthy physical activity in univariate correlations. A similar correlation pattern was present for dietary habits, serving as an explanation as to why these two lifestyle factors are not associated with increased risk of metabolic syndrome in our study.

Modification of cardiovascular risk by adjusting lifestyle habits involving diet, exercise and smoking by self-management programs are not always effective [48-50]. One approach of modifying the cardiovascular risk associated with Type D personality might be modification of health behaviors. However, a complicating factor in implementing self-management programs to address unhealthy lifestyles may be that cardiovascular patients with Type D personality are less likely to consult their physician [6], which makes both physician and the person with Type D personality unaware of their increased risk. Hence investigating the risk of Type D patients in terms of metabolic syndrome and unhealthy lifestyle in a community sample, in which consultation behavior is not an issue, is a first step in unraveling the potential effectiveness of behavioral interventions for cardiovascular disease prevention.

One limitation to our study is that we used self-report measures to assess the components of the metabolic syndrome. It is therefore imperative to establish whether the prevalence of the metabolic syndrome and subcomponents are representative for the Dutch population. The prevalence of the metabolic syndrome in our sample was significantly lower than in others [51,52]. This may largely be due to the use of a proxy self-report measure of metabolic syndrome. We used a more strict three out of four criteria to define metabolic syndrome in order not to overestimate metabolic syndrome prevalence, whereas the IDF definition uses three out of five. However, this reduces the chance of receiving metabolic syndrome diagnosis, e.g. if 25% of a sample has metabolic syndrome, based on 3/5 criteria, we could only detect 20% of this group, based on our 3/4 criteria. The cut-off scores for an increased waist-circumference according to IDF-criteria (55.6%) were comparable with another Dutch sample (57.6% [51], and being overweight (BMI 25-30: 34.7%) or having obesity (BMI > 30: 10.5%) were comparable with the Dutch population (35.7% and 11.1% respectively, http://statline.cbs.nl).

Another limitation of the current study is the cross-sectional nature of the study sample, precluding any causal statements on the progression of metabolic syndrome in people with Type D personality. Strengths include the large sample size and the inclusion of multiple covariates.

## Conclusions

The current study reports a twofold risk of metabolic syndrome associated with Type D personality, independent of socio-demographic, cardiovascular and lifestyle factors.

This relation is likely due to the relation between negative affectivity with hypertension and social inhibition with dyslipidemia. The relation between Type D personality and metabolic syndrome should be evaluated in a clinically measured community sample.

## Competing interests

The authors declare that they have no competing interests.

## Authors' contributions

PMCM and NK conceived of the study, and participated in its design and coordination. PMCM and NK performed the statistical analysis. PMCM, NK and JD drafted the manuscript. All authors read and approved the final manuscript.

## Pre-publication history

The pre-publication history for this paper can be accessed here:

http://www.biomedcentral.com/1471-2458/10/714/prepub
